# CBCT assessment of bone thickness in maxillary and mandibular teeth: an anatomic study

**DOI:** 10.1590/1678-7757-2019-0148

**Published:** 2020-01-31

**Authors:** Olavo César Lyra PORTO, Brunno Santos de Freitas SILVA, Julio Almeida SILVA, Cyntia Rodrigues de Araújo ESTRELA, Ana Helena Gonçalves de ALENCAR, Mike dos Reis BUENO, Carlos ESTRELA

**Affiliations:** 1 Universidade Federal de Goiás Faculdade de Odontologia Departamento de Ciências Estomatológicas GoiâniaGoiás Brasil Universidade Federal de Goiás, Faculdade de Odontologia, Departamento de Ciências Estomatológicas, Goiânia, Goiás, Brasil.; 2 Centro Universitário de Anápolis Departamento de Diagnóstico, AnápolisGoiás Brasil Centro Universitário de Anápolis, Curso de Odontologia, Departamento de Diagnóstico, Anápolis, Goiás, Brasil.; 3 Faculdade São Leopoldo Mandic Faculdade de Odontologia Departamento de Radiologia CampinasSão Paulo Brasil Faculdade São Leopoldo Mandic, Faculdade de Odontologia, Departamento de Radiologia, Campinas, São Paulo, Brasil.

**Keywords:** Cone-beam computed tomography, Fistula, Bone, Endodontics

## Abstract

The site of the sinus tract depends on the rate of resistance against abscess exudate drainage, bone morphology, and distance from the root apex to the outer cortical bone.

**Objective:**

To assess apical bone thickness in buccal and palatal/lingual aspects of maxillary and mandibular teeth, using a high-resolution cone-beam computed tomography (CBCT) system.

**Methodology:**

In total, 422 CBCT examinations were included in the study, resulting in a sample of 1400 teeth. The scans were acquired by PreXion 3D, with a high-resolution protocol. The bone thickness was taken as the distance between the center of the apical foramen and the buccal and lingual/palatal cortical bone. The quantitative variables were expressed as mean values±standard deviation. The independent samples were analyzed using the t-test or the Mann-Whitney test (p<0.05).

**Results:**

The lowest mean value of bone thickness was observed in the buccal cortical bone of the upper canines (1.49 mm±0.86) and in the upper central incisors (1.59 mm±0.67). In premolar teeth, the lowest values were found in the buccal cortical bone of upper first premolars (1.13 mm±0.68). In the posterior teeth, the lowest values were found in the buccal cortical bone of upper first molars (1.98 mm±1.33). In the lower second molar region, the buccal cortical bone (8.36 mm±1.84) was thicker than the lingual cortical bone (2.95 mm±1.16) (p<0.05).

**Conclusions:**

The lowest mean values of bone thickness are in the buccal cortical bone of the maxillary teeth. In the mandible, bone thickness is thinner in the buccal bone around the anterior and premolar teeth, and in the lingual aspect of mandibular molars. All these anatomic characteristics could make the occurrence of the sinus tract more susceptible in these specific regions of the maxillary and mandibular alveolar bone.

## Introduction

Periapical inflammation is a frequent consequence of a chronic infection of endodontic origin. One of the most common inflammatory periapical lesions is the abscess,^1^ which may present a chronic course due to persistence of an endodontic infection, resulting in the formation of a sinus tract.^2^ The sinus tract is a pathologic means of abscess drainage along the path of least resistance through bone and soft tissue, ultimately gaining access to intraoral or extraoral surfaces.^2 , 3^ The site of the sinus tract depends on the rate of resistance against abscess exudate drainage, bone morphology and distance between the root apex and the outer cortical bone^4^ . Therefore, the study of bone thickness in maxillary and mandibular teeth could be a manner to understand the possible drainage routes of a periapical abscess, as well as the epidemiology of the odontogenic sinus tract.

The study of bone thickness in maxillary and mandibular dentition has been gaining attention in Implantology,^5 , 6^ Periodontology,^7^ and Oral Surgery.^8^ The investigation of bone anatomy is important in many branches of Dentistry, influencing surgical planning,^6 , 9^ dental implant rehabilitation outcome,^10^ and selection of the best positioning for skeletal anchorage, which improves orthodontic mechanics.^11^ However, information regarding bone thickness in the apical region of maxillary and mandibular teeth is scarce, though very important for endodontic purposes, specially for the surgical planning in paraendodontic surgery.

Cone-beam computed tomography (CBCT) is an imaging technique that enables the anatomic study of dental and maxillofacial bone structures in cross-sectional high-resolution images *in vivo* .^12 , 13^ This imaging technique also enables linear measurements of dental and bone structures to be performed with accuracy and reliability.^14 - 17^ However, the accuracy of reformatted CBCT images is affected by technical parameters that could depend on the CBCT system, such as nominal resolution, image quality, voxel size, kV, mA, number of basis images, field of view (FOV), and the algorithm of the software used in the acquisition and reconstruction of dimensional measurements.^12 , 18 , 19^ Advanced CBCT systems with high spatial resolution, submillimeter voxel sizes, small FOV, and a smaller focal spot, are considered more accurate in regard to linear measurements.^18^

Although some CBCT studies have been conducted to analyze bone thickness in maxillary and mandibular teeth, the information regarding bone thickness in the apical region have been under-represented. Therefore, the aim of this study was to assess apical bone thickness in the buccal and palatal/lingual aspects of maxillary and mandibular teeth, using a high-resolution CBCT system.

## Methodology

### Sample selection

This study was approved by the Research Ethics Committee of the Institutional Review Board (approval number 7968214.8.0000.5083). CBCT examinations were selected from patients registered in the database of a private radiology clinic (CIRO, Goiânia, GO, Brazil) between January, 2012 and April, 2017. The CBCT scans were performed for various clinical reasons, other than the purpose of this research. The inclusion criteria were: high-resolution images; images from patients older than 18 years; images presenting maxillary or mandibular teeth with a fully formed apex; teeth without calcified root canals; no root canal treatment, post, or crowns; no internal or external root resorption; no history of orthodontic treatment; no developmental disorders; and no periapical diseases. Impacted teeth and supernumeraries were excluded. This study included the measurements of the bone around all teeth, except the third molars. The sample size was calculated according to a pilot study that determined 90% of the bone thickness presenting 8% variation (more or less) depending on which tooth was examined. At a power of 80% and a significance level of 5%, a sample of 54 roots would be necessary for each group, totaling 756 teeth. In this study, 1400 teeth were included, which ensured a lower margin of error and higher reliability of results. In total, 422 CBCT examinations were included in the study, resulting in a convenience sample of 1400 teeth.

### CBCT image acquisition

The scans were acquired by PreXion 3D (TeraRecon Inc., San Mateo, CA, USA), with the following exposure protocol: 60x56 mm FOV, 33.5 seconds of exposure time, 90 kVp, 4 mA, thickness of 0.100 mm, voxel size of 0.100 mm and 1024 basis images. The images were analyzed using PreXion 3D Viewer software (TeraRecon Inc., Foster City, CA, USA) on a workstation with Windows 7 Professional SP-2 (Microsoft Corp, Redmond, WA, USA), equipped with an Intel I7 1.86 Ghz-6300 processor (Intel Corp, Santa Clara, CA, USA), NVIDIA GeForce 1070 turbocharged video card (NVIDIA Corporation, Santa Clara, CA, USA), and a high-resolution EIZO-Flexscan S2000 monitor with a resolution of 1600x1200 pixels (EIZO NANAO Corp, Hakusan, Japan).

### Image analysis

**Figure 1:**
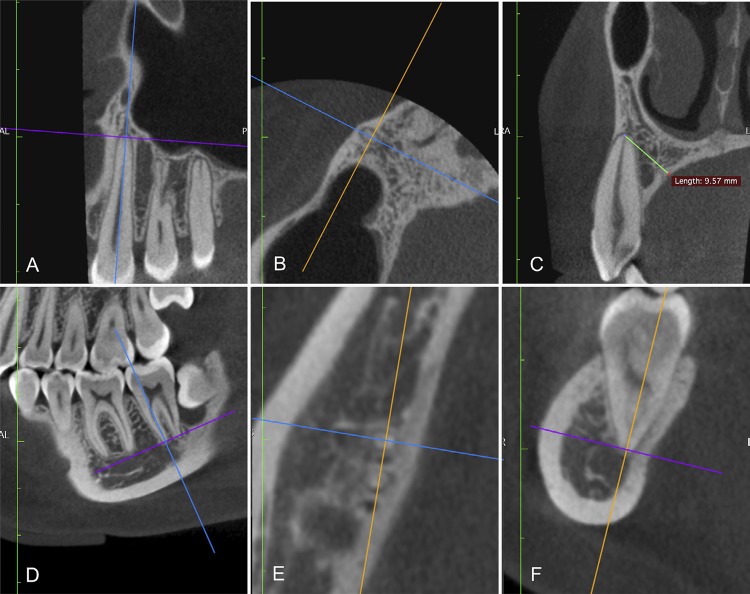
CBCT images in the sagittal, axial, and coronal planes (A-F). Standard reference for the location of the apical foramen was the main root canal. Axial navigation was used for each root individually

**Figure 2:**
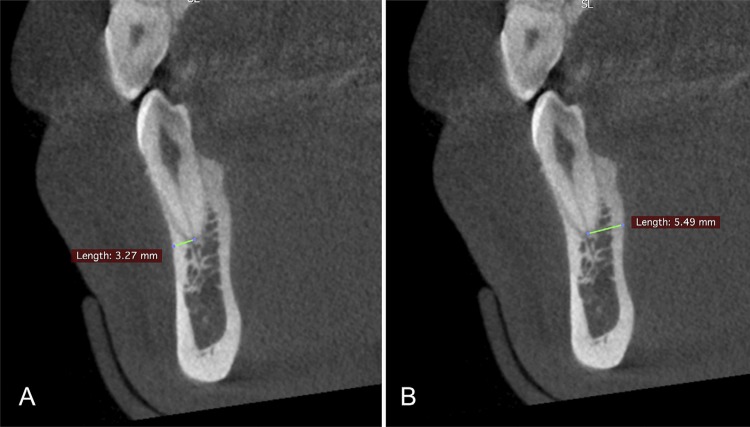
The smallest measurement for the anterior teeth was found in the sagittal plane (buccal and lingual bone measurements)

**Figure 3:**
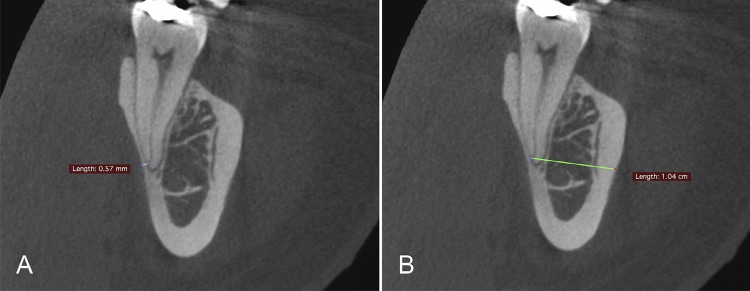
The smallest measurement for the posterior teeth was found in the coronal plane (buccal and lingual bone measurements)

The map-reading dynamic feature of the CBCT was applied as described previously,^20^ to improve the visualization and identification of the apical foramen and bone walls. The bone thickness was considered as the distance between the center of the apical foramen, the buccal and the lingual/palatal cortical bones, and was determined by the CBCT images in the axial, sagittal, and coronal planes ( Figure 1 ). The smallest measurement of the anterior teeth was defined in the sagittal plane ( Figure 2 ), and the posterior teeth, in the coronal plane ( Figure 3 ). The Figures 2 and 3 were visualized using a new CBCT software program named e-Vol DX (CDT Software, Bauru, SP, Brazil).^12^

The bone thickness was measured using the PreXion 3D Viewer software (TeraRecon Inc., Foster City, CA, USA). The standard reference for the location of the apical foramen was the main root canal. Axial navigation was used for each root individually. In the upper molars, axial navigation began in the mesiobuccal root (MB), followed by analysis of the distobuccal (DB) and palatal roots (PR). In the lower molars, the navigation started in the mesial root (M), followed by analysis of the distal root (D). In the presence of fused roots, the axial navigation analyzed the two roots concomitantly. Two observers, specialists in dental radiology with more than 10 years of experience, analyzed all the images. When differences were found, a consensus was reached by discussion of each case between the two examiners.

### Statistical analysis

The mean and standard deviation of the quantitative variables were obtained. Data normality was assessed by the Kolmogorov-Smirnov test. The variance of the groups was assessed by the Levene’s Test. Comparison analysis of independent samples was assessed by the t-test for independent samples — used for data with normal distribution and for groups with statistically homogeneous variances — or by the Mann-Whitney test for data that did not present normal distribution and for groups presenting statistically heterogeneous variances. Fisher’s exact test was used to examine associations between categorical variables. Pearson’s correlation coefficient was calculated to examine associations between quantitative variables. The level of significance was set at α=0.05. Statistical analysis was performed using Statistical Package for Social Sciences software, version 20 (SPSS, Chicago, IL, USA).

## Results

A total of 422 CBCT examinations from patients of a private radiology clinic composed this research; 394 were women and 28 were men, with a mean age of 44.46 years. These examinations resulted in a sample of 1400 teeth distributed as follows: Maxillary teeth: central incisors, n=100; lateral incisors, n=100; canines, n=100; first premolars, n=100; second premolars, n=100; first molars, n=100; second molars, n=100. Mandibular teeth: central incisors, n=100; lateral incisors, n=100; canines, n=100; first premolars, n=100; second premolars, n=100; first molars, n=100; and second molars, n=100.

The mean buccal and lingual/palatal bone thickness in maxillary and mandibular anterior teeth and their descriptive statistics with maximum and minimum values are presented in Table 1 . In anterior teeth, the lowest mean value of bone thickness was observed in the buccal cortical bone of the upper canines (1.49 mm±0.86) and in the upper central incisors (1.59 mm±0.67). The palatal aspects of the upper canines (8.63±2.08 mm) and of the upper central incisors (7.07 mm±1.96) presented the highest mean values.

**Table 1:** Buccal and lingual/palatal bone thickness of maxillary and mandibular anterior teeth a,b, in the sagittal plane

Tooth	Buccal cortical bone thickness	N	Min	Max	95%CI	Lingual/palatal cortical bone thickness
(n=600)						

UCI	1.59±0.67	100	0.33	3.68	1.45-1.72	7.07±1.96
ULI	2.30±1.20	100	0.76	6.67	2.07-2.54	5.28±1.35
UC	1.49±0.86	100	0.16	4.84	1.32-1.66	8.63±2.08
						
LCI	2.72±1.30	100	0.46	6.05	2.46-2.98	3.89±1.15
LLI	3.06±1.29	100	0.56	5.98	2.81-3.32	4.01±1.35
LC	3.43±1.31	100	0.70	6.77	3.17-3.69	4.78±1.64

X ®: mean. SD: standard deviation. a: t- test for independent samples. b: Mann-Whitney test. UCI=Upper central incisors. ULI=Upper lateral incisors. UC=Upper canines. LCI=Lower central incisors. LLI=Lower lateral incisors. LC=Lower canines

Table 2 presents the mean values and the descriptive statistics with maximum and minimum values of buccal and lingual/palatal bone thickness in maxillary and mandibular premolar teeth. The smallest bone thickness was found in the buccal cortical bone, related to the buccal roots of the upper first (1.13 mm±0.68) and second (2.20 mm±1.21) premolars. The lingual/palatal cortical bone of the palatal roots of upper first (8.07±1.63) and second (7.62 mm±1.84) premolars was found to be thicker than the buccal cortical bone.

**Table 2:** Buccal and lingual/palatal bone thickness of maxillary and mandibular premolar teeth a,b, in the coronal plane

Tooth	Buccal cortical bone thickness	N	Min	Max	95%CI	Lingual/palatal cortical bone thickness	N	Min
(n=400)								

UFP								
BR	1.13±0.68	100	0.13	3.31	1.00-1.27	8.07±1.63	100	4.36
PR	4.47±1.85	86	1.29	13.4	4.07-4.86	4.52±1.51	86	1.63
USP								
BR	2.20±1.21	97	0.19	5.65	1.96-2.44	7.62±1.84	97	0.00
PR	3.86±1.74	36	0.00	7.96	3.27-4.45	5.82±1.59	36	3.22
LFP	3.27±1.04	100	0.89	6.13	3.07-3.48	5.58±1.66	100	1.47
LSP	3.65±1.35	100	0.92	7.48	3.38-3.92	5.46±1.84	100	1.56

X ®: mean. SD: standard deviation. a: t-test for independent samples. b: Mann-Whitney test. UFP=Upper first premolars. USP=Upper second premolars. LFP=Lower first premolars. LSP=Lower second premolars. BR=Buccal root. PR=Palatal root

The mean values of buccal and lingual/palatal bone thickness in maxillary and mandibular posterior teeth and their descriptive statistics with maximum and minimum values are presented in Table 3 . In the posterior teeth, the lowest mean values of bone thickness were found in the buccal cortical bone of the mesiobuccal root of the upper first molars (1.98 mm±1.33). In the lower second molar region, the buccal cortical bone (8.36 mm±1.84) was thicker than the lingual cortical bone (2.95 mm±1.16). All the teeth groups — anterior and posterior teeth — presented significant differences in bone thickness, comparing the buccal with the lingual/palatal cortical bones (p<0.05) (Tables 1, 2 and 3). Age was not significantly associated with the buccal and lingual/palatal bone thickness in this population (r=0.377, p=0.089).

**Table 3:** Buccal and lingual/palatal bone thickness of maxillary and mandibular posterior teeth a,b, in the coronal plane

Tooth	Buccal cortical bone thickness	N	Min	Max	95%CI	Lingual/palatal cortical bone thickness	N	Min	Max	95%CI	p
(n=400)											

UFM											
MBR	1.98±1.33	100	0.10	5.98	1.72-2.25	11.91±1.68	100	8.11	16.77	11.57-12.24	0.000**
MPR	3.10±1.35	76	0.10	6.17	2.80-3.41	10.56±1.81	76	7.37	16.77	10.15-10.98	0.000*
DBR	2.07±1.45	100	0.06	5.79	1.79-2.36	12.35±1.98	100	1.14	17.00	11.96-12.74	0.000**
PR	11.92±2.38	100	1.08	17.44	11.44-12.39	2.84±1.16	100	0.61	6.48	2.61-3.07	0.000**
USM											
MBR	4.48±1.85	100	0.79	8.79	4.11-4.85	8.74±2.47	100	2.58	14.70	8.25-9.23	0.000*
MPR	4.89±1.48	34	1.41	7.94	4.37-5.40	8.46±2.56	34	1.24	13.60	7.57-9.35	0.000*
DBR	3.51±2.15	89	0.11	12.80	3.06-3.96	9.74±2.41	89	3.29	14.60	9.24-10.25	0.000**
PR	10.39±2.42	92	2.61	15.14	9.88-10.89	2.82±1.86	92	0.48	14.60	2.43-3.20	0.000**
LFM											
MBR	4.45±1.46	100	1.71	8.33	4.16-4.74	6.49±1.87	100	2.59	11.00	6.12-6.86	0.000**
MLR	5.43±1.41	100	2.48	8.77	5.15-5.71	5.63±1.88	100	1.00	9.90	5.26-6.00	0.397*
DMR	5.91±1.64	100	1.84	10.50	5.58-6.24	5.44±1.82	100	1.30	9.77	5.08-5.80	0.056*
DLR	6.46±1.86	24	2.77	10.90	5.68-7.25	4.68±2.16	24	1.00	9.20	3.77-5.59	0.004*
LSM											
MBR	7.73±1.83	100	3.32	13.70	7.37-8.10	3.46±1.24	100	1.04	7.26	3.21-3.70	0.000*
MLR	8.36±1.84	80	4.4	13.70	7.95-8.77	2.95±1.16	81	0.46	6.12	2.69-3.20	0.000*
DBR	8.01±1.91	95	3.25	15.20	7.62-8.39	3.08±1.13	94	0.57	5.81	2.85-3.31	0.000**
DLR	6.65±4.47	4	0.00	9.31	0.45-13.76	2.19±1.57	4	0.00	3.67	0.31-4.68	0.191**

X ®: mean. SD: standard deviation. a: t-test for independent samples. b: Mann-Whitney test. UFM=Upper first molars. USM=Upper second molars. LFM=Lower first molars. LSM=Lower second molars. MBR=Mesiobuccal root canal. MPR=Mesiopalatal root canal. DBR=Distobuccal root canal. PR=Palatal root canal. MLR=Mesiolingual root canal. DLR=Distolingual root canal

## Discussion

Bone thickness could influence the drainage routes of the odontogenic periapical abscess, and consequent formation of the sinus tract.^21^ Therefore, the study of maxillary and mandibular apical bone thickness could be an important aid to understand the formation of sinus tract, and to consolidate the data published about its epidemiology and diagnosis in endodontics. In this respect, our study aimed to assess the apical buccal and palatal/lingual bone thickness in maxillary and mandibular teeth, using a high-resolution CBCT unit.

In this study, the lowest mean values of apical bone thickness were found in the buccal cortical bone of the maxillary teeth, especially in the anterior canines, central incisors, first premolars, and first molars. These results corroborate those of epidemiological studies, which have found a higher prevalence of the odontogenic sinus tract in the maxilla,^2 , 4^ particularly in the buccal aspect of upper incisors, upper premolars, and molars.^2^ The thin cortical bone found in the buccal aspect of maxillary teeth could contribute to a higher prevalence of the sinus tract in these locations, for the distance between the tooth apices and the external cortical surface in these regions is usually short, and the sinus tract typically follows a path of least resistance through the alveolar bone.^3^ In fact, the palatal alveolar bone in the apical region appears to be thicker than the buccal bone, as observed in this investigation, and is generally more compact,^3^ thus explaining why it is rare to have a palatal sinus tract.^2 , 4 , 22^ This study found that the palatal root of the upper second premolars is closer to the buccal cortical bone than the palatal cortical bone itself. This may explain why the sinus tract in maxillary teeth is often detected in the buccal alveolar bone.

Regarding the mandible, the bone thickness was thinner in the buccal bone around the anterior and premolar teeth. These findings corroborate published epidemiological data that indicate a prevalence of the sinus tract in the buccal aspect of the mandible.^2 , 4 , 22^ Curiously, the occurrence of a lingual sinus tract is typically observed in mandibular molars.^22^ Our findings may explain this occurrence, since we found lower mean values of lingual bone thickness in the apical region of the first and second mandibular molars. In some instances, this anatomic characteristic could support the occurrence of the sinus tract in the lingual aspect of the mandibular bone.

Zahebi, Mostafavi, Lotfirikan^23^ (2018) recently investigated the buccal and lingual bone thickness of mandibular premolar and molar roots using CBCT imaging. They found lower values of lingual bone thickness in mandibular molars region. Aindin and Bulut^24^ (2019) found lower values of lingual bone thickness in mandibular molar in a study which proposed to investigate the buccal and lingual bone thickness overlying mandibular posterior teeth. Although these two studies had similar results to those found in this study, the comparison between their and our results could be inappropriate, for Zahebi, Mostafavi, Lotfirikan^23^ (2018) assessed buccal and lingual bone thickness in the largest size of the axial plane in CBCT images, and Aindin and Bulut^24^ (2019) measured buccal and lingual bone at 3 mm apical resection level. In our study, we considered the distance between the center of the apical foramen, buccal and lingual/palatal cortical bones as bone thickness, in the axial, sagittal and coronal planes of CBCT images. Our method was conceived to mimic the sinus tract pathway.

Bone thickness acts as an important factor influencing the development of the sinus tract in bone,^25^ associated with dental caries and trauma incidence. These adverse factors may influence the prevalence of periapical abscess, and consequential prevalence of odontogenic sinus tract in specific dental groups.^4 , 26^ This finding is based on the premise that the most common initiating factors of a periapical abscess have low incidence in teeth where the sinus tract is very uncommon, e.g., maxillary and mandibular canines. According to Slutzky-Goldberg, et al.^22^ (2009), there are some reasons why canines are not usually involved in the sinus tract. The authors believe that the sinus tract is less common in the canines, for their apices are embedded in a thick cortical bone. They also suggest that canines are less commonly affected by caries or trauma, thus representing another relevant factor for the sinus tract to be uncommon in this specific tooth. In this investigation, some of our results are in line with the convictions held by Slutzky-Goldberg, et al.^22^ (2009), since we found that lower canines presented a thicker buccal cortical bone, in comparison with other lower anterior teeth. However, we observed that the buccal bone thickness of upper canines is very thin, having a mean value of 1.49 mm. This suggests that the buccal bone of upper canines offers less resistance to the spreading of inflammatory content of a periapical abscess, thus representing a relevant factor supporting the formation of a sinus tract. Thus, it is plausible to assume that the presence of initiating factors, such dental caries and trauma, may influence sinus tract prevalence in specific teeth. Additionally, it has been postulated that sinus tract formation depends on other factors, such as seriousness and virulence of microorganisms involved in a periapical abscess.^27^

Most of the studies available about the sinus tract indicate a significant prevalence of this condition in endodontically treated teeth, representing an important sign of failure in endodontic therapy.^2 , 4 , 22^ This presents what can be considered a critical consideration regarding the presence of the sinus tract in previously treated endodontic teeth. The prevalence of this sign of therapeutic failure is higher in posterior teeth,^4^ probably due to the anatomic complexity of their root canal system, which can affect their cleaning, shaping and obturation, and which can consequently influence endodontic therapeutic success.^17 , 28^ In this study, it was observed that some of the root canals of posterior teeth are very close to the cortical bone; this could favor the drainage of inflammatory content through the bone. In addition to the above-mentioned factors, bone thickness suggests to contribute in different manners to the prevalence of the sinus tract in posterior teeth; however, the exact relevance of each of these factors in sinus tract pathogenesis is unknown.

One of the limitations of this study was that bone measurements by CBCT may have been influenced by the root angulation of the teeth analyzed, possibly leading to divergence among individual members of the population. According to Srebrzyńska-Witek, et al.^29^ (2018), the thickness of buccal spongious bone increases around anterior teeth — along with the inclination of the dental axis — as the thickness of lingual spongious bone decreases.^29^ However, in their study no assumption was made regarding the cortical apical bone, thus leaving uncertain the influence of the inclination of the tooth root in this specific region of the alveolar bone. We also believe that sex and age may be factors that influence the thickness of the alveolar bone. However, in our sample there was a predominance of women, with mean age above 35 years, hindering the verification of these differences.

In this study, maxillary and mandibular bone thickness were analyzed by a high-resolution CBCT system, selected due to its ability to represent bony structures in a highly accurate way.^30^ In most clinical applications, CBCT is considered an accurate imaging examination providing reliable information with respect to linear measurements.^14 , 31^ It is recognized that some technical parameters of the CBCT, such as spatial resolution, voxel size, FOV, focal point, number of basis images and the reconstruction algorithm, can influence in the dimensional measurements obtained by this imaging examination.^32^ In this study, CBCT system images were used with high spatial resolution, submillimeter isotropic voxel (0.100 mm), small FOV (60x56 mm), small focal spot (0.3 mm) and 1024 basis images, with the objective of reducing the influence of these parameters on the linear measurements of bone thickness. It should be highlighted that the reconstruction of images and the linear measurements were performed in native CBCT system software, respecting the reconstruction algorithms determined by the manufacturer. The combination of all these technical parameters produced a more accurate CBCT image in regard to linear measurements.^12^ Consequently, this high-resolution CBCT system could be considered reliable in defining alveolar bone thickness.

## Conclusions

The lowest mean values of apical bone thickness were found in the buccal cortical bone of the maxillary teeth, especially in anterior canines, central incisors, first premolars and first molars. In the mandible, the bone thickness in the buccal bone is thinner around the anterior and premolar teeth, and in the lingual aspect of the apical region of mandibular first and second molars. All these anatomic characteristics could increase the occurrence of the sinus tract in these specific regions of maxillary and mandibular alveolar bone.
